# The effects of NONRATT008453.2 on autophagy in genital tubercle fibroblasts of rats with hypospadias induced by dibutyl phthalate

**DOI:** 10.1002/bdr2.1863

**Published:** 2021-01-15

**Authors:** Xiao Feng, Enfu Huang, Yuanyuan Gao, Ya Zhang, Yun Zhou

**Affiliations:** ^1^ Department of Clinical laboratory Children's Hospital of Soochow University Suzhou P. R. China; ^2^ Section of Pediatric Urology Children's Hospital of Soochow University Suzhou P. R. China; ^3^ Central Laboratory of Pediatric Research Institute Children's Hospital of Soochow University Suzhou P. R. China

**Keywords:** autophagy, dibutyl phthalate, hypospadias, long noncoding RNAs, rat

## Abstract

**Background:**

Hypospadias is a common birth defect that might be caused by inadequate fusion of the urethral folds in the process of male external genital development. We intended to discover the crucial long noncoding RNAs (lncRNAs) regulating autophagy from the gene expression profile of the genital tubercle (GT) of dibutyl phthalate (DBP) induced hypospadiac rats.

**Methods:**

Whole transcriptome resequencing was used to determine the expression of the total RNA in GTs and cultured fibroblasts obtained from GTs of DBP‐induced hypospadiac male rat fetuses. Autophagosomes and autolysosomes were examined under a transmission electron microscope after overexpression of lncRNA NONRATT008453.2 in the fibroblasts by adenovirus transfection. Finally, the protein expression levels of Atg5, Beclin‐1, Atg7, and the LC3A/B‐II:LC3A/B‐I ratio were detected in the fibroblasts by western blotting.

**Results:**

NONRATT008453.2 suppressed autophagy by promoting the expression of Atg7, but inhibited the expressions of Atg5, Beclin‐1, and the LC3A/B‐II:LC3A/B‐I ratio in the GT fibroblasts.

**Conclusions:**

NONRATT008453.2 may have an influence on autophagy in the fibroblasts of the GT in DBP‐induced hypospadiac rats.

## 1 INTRODUCTION

Hypospadias is a common genital anomaly with an estimated incidence of 1:300 male live births (Blaschko, Cunha, & Baskin, 2012). Although the precise pathogenetic mechanism of hypospadias is not clear, several factors are believed to play a role in its causation, including genetic, endocrine, and environmental factors (Kalfa, Philibert, & Sultan, 2009). Environmental endocrine disruptors (EEDs), such as dibutyl phthalate (DBP), are widely used in the plastics industry and have been implicated in this developmental anomaly. The disruption of androgen homeostasis and steroid hormone receptors may play a crucial role in the onset of hypospadias (Lee & Koo, 2007). The androgen signaling pathway is closely associated with autophagy, which affects cell survival in regulating various pathological processes (He & Klionsky, 2009).

Long noncoding RNAs (lncRNAs) are a family of nonprotein‐coding RNAs greater than 200 nucleotides in length (Li, Li, Zhang, & Zhou, 2017; Li, Li, Zhang, & Zhou, 2017). Over the past decades, increasing evidence has indicated that lncRNAs participate in autophagy regulatory networks (Yang, Wang, Shen, Feng, & Jin, 2017). It is, therefore, imperative to investigate the role of specific lncRNAs in regulating autophagy during the process of formation of the penile urethra.

In order to know what leads to hypospadias in the critical period of urethral closure, we hypothesized EEDs lead to abnormal urethral closure by affecting autophagy, and sought to find out whether lncRNAs participate in and regulate this process. There are not many studies on lncRNA involved in autophagy regulation, and the regulatory mechanism is not very clear. To find out the critical lncRNA and explore its effect on autophagy is of great significance to the prevention and treatment of hypospadias.

## 2 MATERIALS AND METHODS

### 2.1 Hypospadias specimens

Adult Sprague–Dawley (SD) rats (age: 2 months) were purchased from the University Experimental Animal Center and housed under controlled conditions (12 hr light–dark cycle; temperature: 22 ± 4°C; humidity: 40–80%). The animals were provided ad libitum access to fresh tap water and standard rat chow. After 2 weeks of acclimatization, 25 virgin female rats (body weight: 240 ± 10 g) were mated overnight with male rats in a 1:1 ratio. The presence of a vaginal plug on the second morning after mating was considered indicative of gestation day 1 (GD1). Pregnant rats were then randomly divided into two groups: DBP‐treated and controls. In the DBP‐treated group, the pregnant rats (*n* = 14) were administered DBP (Sigma Research, Inc., Richland, WA, USA) dissolved in corn oil by oral gavage at a dose of 800 mg/kg/day from GD 13 to GD 18. In the control group, the pregnant rats (*n* = 11) were administered an equal volume of corn oil only (Sigma Research, Inc., Richland, WA, USA). On GD19, fetuses were harvested by cesarean section from pregnant rats and examined for the occurrence of hypospadias using a dissecting microscope. There were 84 genital tubercles (GTs) collected from male fetuses in DBP‐treated group and 67 GTs collected from the control group. Hypospadias is defined as the presence of any one or more of the following three associated anomalies: (a) ectopic urethral meatus; (b) penile curvature (chordee); and (c) ventral foreskin deficiency (Mouriquand, Persad, & Sharma, 1995). The rats were sacrificed by cervical dislocation. All procedures and protocols carried out were approved by the Experimental Animal Ethics Committee of Soochow University, and all applicable institutional and governmental regulations concerning the ethical use of animals were followed. Paraffin sections of GTs of four male hypospadias rats from different litters in the DBP‐treated group and four male rats from different litters in the control group were made and stained with hematoxylin and eosin (H&E) to examine the pathological features. Six GTs of hypospadiac fetuses in DBP‐treated group and six GTs of male fetuses from normal control (three litters from each group, and two GTs from each litter) were harvested and subjected to whole transcriptome resequencing. The remaining GT tissues of male fetuses from DBP‐treated group and normal control group were used for primary cell culture of fibroblasts and subsequent molecular biology studies.

### 2.2 RNA‐sequencing and bioinformatics analysis

Selected GTs were lysed with Trizol and total RNA was extracted according to the instructions accompanying the test kits (Promega, Madison, WI，USA). We measured the concentration and the quality of the total RNA using a spectrophotometer (Thermo Fisher Scientific, Inc., Waltham, MA, USA) and gel electrophoresis. An RNA‐sequencing library was conducted and the whole transcriptome sequencing was performed using Illumina HiSeqTM 2,500 sequencers (Illumina, Inc, San Diego, CA, USA) after the testing was qualified by a biological analyzer (Agilent Technologies, Santa Clara, CA, USA).

For the function prediction of lncRNAs, we adopted a method originally demonstrated on paper. In brief, we first calculated co‐expressed mRNAs for each differentiated lncRNA (correlation *p* < .05), and then conducted a functional enrichment analysis of this set of co‐expressed mRNAs. The enriched functional terms were used as the predicted functional term of a given lncRNA. We screened out the lncRNAs that were related to autophagy, and then selected the different lncRNAs with foldchange ≥2. Finally lncRNAs (foldchange ≥2) with high expression levels were identified and verified by quantitative reverse transcription PCR (qRT‐PCR) using the SYBR Green method (Qiagen, Valencia, California, USA). The primers of the lncRNAs and the internal control gene ACTB are shown in Table 1.

**TABLE 1:** LncRNAs' primer sequences

Gene name	Forward primer (5′ → 3′)	Reverse primer(5′ → 3′)
NONRATT009918.2 NONRATT024766.2 NONRATT004969.2 NONRATT008453.2 NONRATT031494.1 NONRATT028181.2 NONRATT028873.2 NONRATT024624.2 NONRATT010304.2 ACTB	GACATTGGGTCAGGGACT AGATCAAGATCAGCTTCACTCA GCCTAACAGATAGCTGGCA AATCCACTTGATCTTCCACTG GAAGAGATTGAACTCCTCCG TTCATGGTGGTGTAGGTGATAG TGACAAGCATAATCTCTGGTCT GAACTCAAAGTCGTCGCCCTA GTGAGCACTCAACACT TGAAT CCACCATGTACCCAGGCATT	GGGTCCCTGTATCCACAAA ACTAAAGACACGACAATCCAT CAGGTTCTGACTCACTTGG CCATCCTCAAAGACAAGGAAC ACCGAACAGCTTCTTGATG AAAGAACACAGGAGAGCATT GCTGAAACATTATACAGCACGA TCCTCATGTCCTCTCTTCTTCA TTACCCAAAGACGAAACCCT CGGACTCATCGTACTCCTGC

### 2.3 Primary cell culture and verification

Under aseptic conditions, we cut the GTs of hypospadiac male fetal rats from the same litters into 1–2 mm^3^ tissues in DMEM/F12 medium without serum. The number of GTs depends on the number of male fetuses with hypospadias in a litter. We transferred these tissues into culture bottles, 10 pieces in each. We kept the culture bottles inside an incubator (37°C, 5% CO_2_) for about 30 min and then added DMEM/F12 medium with 10% fetal bovine serum (FBS). The tissues were cultured in an incubator and the medium was changed every 1 or 2 days.

The tissues were removed after the cells migrating from the tissues covered 90% of the bottom of the culture bottle. The cells were then dissociated with 0.125% trypsin for 2–3 min and then treated with a trypsin inhibitor. Subsequently, the cells were centrifuged at 12000*g* for 5 min at 4°C and cultured in DMEM/F12 with 10% FBS in an incubator. The medium was exchanged approximately every 2 days based on the culture state. The fibroblasts were screened by 0.125% trypsin digestion to inhibit the growth of epithelial cells.

The fibroblasts were seeded in a six‐well cell culture plate and 0.2% Triton X‐100 was added to dissociated specific glycoproteins, and blocked using 5% skimmed milk in 0.01 M PBS, then incubated overnight with antibody vimentin (V9) (mouse monoclonal IgG1) (Santa Cruz Biotech, Inc, CA., USA) and diluted at 1:50 ratio in PBS at 4°C. One day later, the cells were incubated with rabbit anti‐mouse IgG–HRP (Santa Cruz Biotech, Inc, CA, USA) and diluted at 1:50 ratio for 2 hr at room temperature. Finally, they were observed and photographed under a fluorescence microscope (Olympus, Tokyo, Japan).

### 2.4 Transfection

A recombinant adenovirus vector with overexpression of the specific lncRNA was established. The carrier vector was GV135 and the packaging plasmids were pBHG lox ΔE1 and 3 Cre (Microbix Biosystems, Ontario, Canada). The NONRATT008453.2‐specific overexpression vector (GV135) and empty vector (negative control) were prepared by Shanghai Genechem Co., Ltd., China. These recombinants were transfected into fibroblasts using a Lipofectamine 2000 reagent (Invitrogen, Carlsbad, CA, USA) according to the manufacturer's instructions. The sequence of NONRATT008453.2 plasmid was: CCGATCTGGCAGCTGAACTAGCTCATCCAGTTTGAGGGGACAGCAGGTGGCCAGAGGGGCAGAGCCCATCTGAATGCCGACTTCCCTGAAGCTGGAAGCTTTAATCCACTTGATCTTCCACTGCCGGCTTAAGGCTCAGCGAGGGGCAGAGGTGCTGGCCTAACTTTGCGTTGGTGTGTTGTTCCTTGTCTTTGAGGATGGTGTGGGGAGGGGGTGGCCACCGTATGTTTGGAAGGTCTCTGCAATAGGCTGCTGCCAGGATTCATTCAGCTCTTTCAGCCCCATCTTGTGAACCTCTGCATGCCACTAGCTTTCCAATTTGTCCCCTCTCAGTGTCTGAGTTGGAAATGGCCTTGAACATGGACCCAGCTGCTTTTCAGACTTCATACTGTGCTGAGAAGCCAAAGCTAGAAGACAAGAATCTTATTTTCTTCTGTCAGATGGGCAGGCGGGGCCTCCAGGCCACACAGCTGGCACAAGGTCTTGGATACACAGGGGCTCGAAACTATGCCGGGGCCTATAAGGAATGGCTGGAGAAAGAGGGCTAAATAGAAGGCTTACTGATTTGTGATTGCTCTCATGGCCACCTTGGTGTGCTCGGCAAATCCTTGTAGTGTTTTACACATGACAAATTTAAATAAAAAGAGCATTTAATG.

### 2.5 CCK‐8 proliferation assay

Fibroblasts of 3 × 10^3^ were seeded into each well of 96‐well plate and further grew in 100 μl complete medium for 24, 48, and 72 hr. Then, 10 μl of CCK‐8 (Bimake, Beijing, China) solution was added to each well and the cells were incubated for 1 hr. Finally, absorbance at 450 nm was detected using a microplate reader (Bio‐Rad Lab, Richmond, CA, USA).

### 2.6 RT‐PCR

Total RNA was extracted from the cultured cells using TRIzol (Invitrogen, Carlsbad, CA, USA). The RNA was reverse‐transcribed to cDNA by gradient thermal cycler (Applied Biosystems, lnc, MA, USA) and then qRT‐PCR was performed using a SYBR premix real‐time PCR reagent (Roche, Diagnostics, Basel, Switzerland). The relative quantification was analyzed using the comparative threshold cycle (2 − ΔΔCT) method by real‐time fluorescence quantitative analyzer (Roche, Basel, Switzerland). The primers for NONRATT008453.2 were as follows: forward primer (5′ → 3′) AATCCACTTGATCTTCCACTG, reverse primer (5′ → 3′) CCATCCTCAAAGACAAGGAAC.

### 2.7 Transmission electron microscopy

A transmission electron microscope (TEM) (Jeol, JEM‐1400 PLUS, Tokyo, Japan) was used to observe autophagy in the fibroblasts after infecting them with adenovirus constructs containing NONRATT008453.2. The specimens were dehydrated by passage through graded alcohol series and acetone, embedded in Epon812, and then cut into ultrathin sections (50–70 nm) with an ultramicrotome (Leica, Wetzlar, Germany). The sections were stained with both 2% uranyl acetate and lead citrate and then examined under the TEM to identify autophagosomes and autolysosomes based on characteristic double‐or multiple‐membranes vacuoles wrapping cellular constituents. A total of 400 cells in randomly selected fields in each specimen were counted.

### 2.8 Western blotting

The total protein products obtained from cultured cells were lysed with RIPA buffer (Beyotime, Shanghai, China). The protein concentration for each sample was measured using an enhanced BCA protein assay kit (cat. no. p0010s; Beyotime, Shanghai, China). Denatured protein products of 40 μg were separated by 12% SDS‐polyacrylamide gel electrophoresis and then electro‐transferred to PVDF membranes. After blocking the membranes with 5% fat‐free milk at room temperature for 1 hr, the membranes were incubated overnight with an autophagy antibody sampler kit (cat.no.4445; Cell Signaling, Danvers, MA, USA) targeting Atg5, Beclin‐1, Atg7, LC3A/B, with a dilution of 1:1000 at 4°C. Subsequently, the membranes were incubated with horseradish per‐oxidase (HRP)‐conjugated secondary antibodies (anti‐rabbit) with a dilution of 1:2000 for 2 hr. The protein bands were detected using a chemiluminescent detection system (Bio‐Rad Lab, Richmond, CA, USA).

### 2.9 Statistical analysis

Data were analyzed using SPSS 21.0 software (IBM SPSS, Armonk, NY, USA) and presented as mean ± standard deviation (SD). Two groups were compared using the unpaired, two‐tailed Student's *t*‐test. ANOVA was used for multi‐group comparisons. *p* < .05 were considered indicative of statistical significance.

## 3 RESULTS

### 3.1 Effects of DBP on male rat fetuses

**FIGURE 1:**
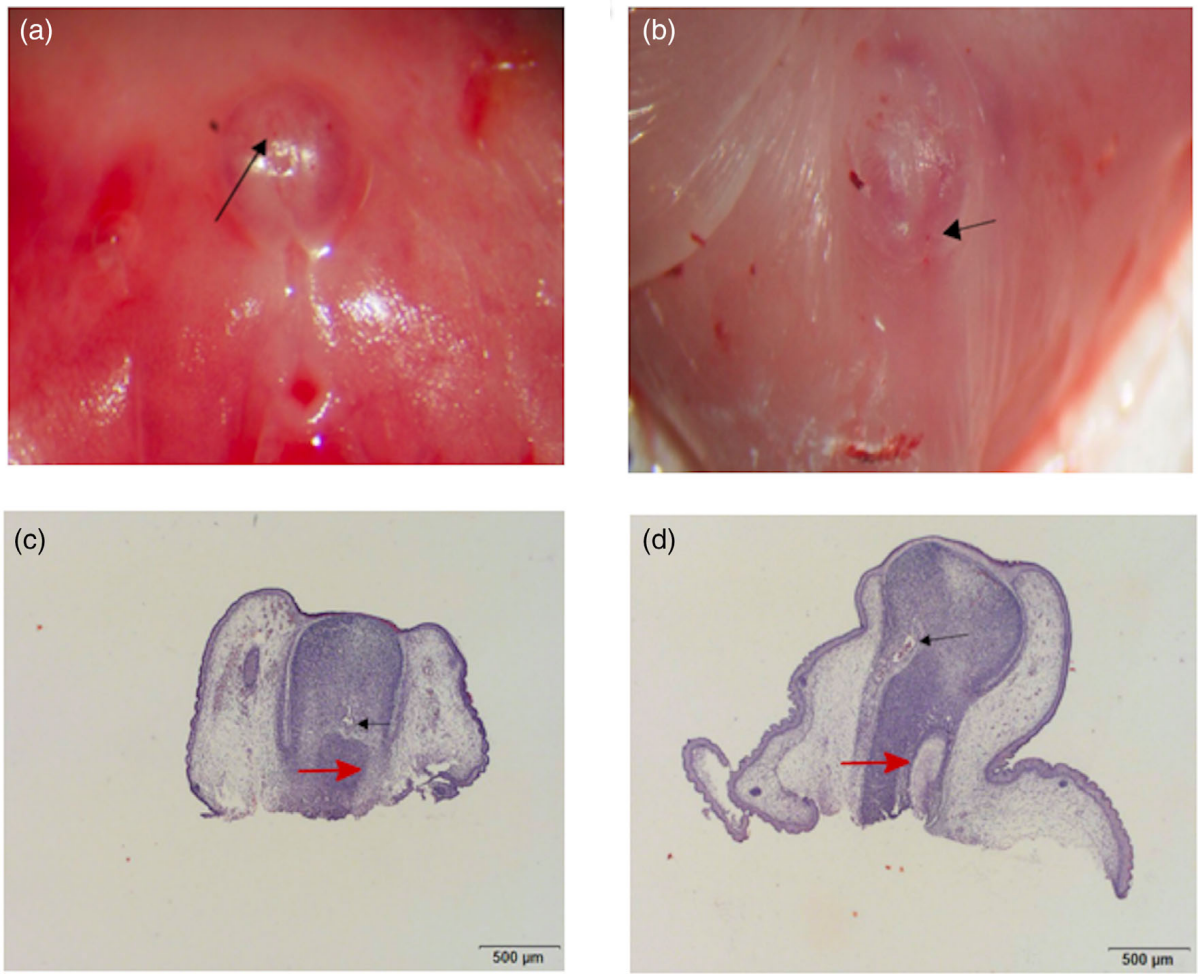
Morphology and histology of GT of male rat fetuses at GD19. In normal male rat fetuses (*n* = 4), the urethral orifice is at the tip of the GT (arrow) (a).The GT has a well‐developed urethra (black arrow) with fusion of urogenital folds (red arrow) (*n* = 4) (c). In male rat fetuses with hypospadias (*n* = 4), the urethral opening is on the body of the genital tubercle (arrow) (b). The urethral opening was blurred (black arrow) and the urogenital folds were not well fused (red arrow) (n = 4) (d) (magnification: ×40)

Fourteen pregnant rats were exposed by DBP from GD13 to GD18, and at GD19, 181 fetuses were harvested from the uterus for examination under a dissecting microscope. Eighty‐four male fetuses were identified in the DBP treatment group, of which 41were affected with hypospadias (incidence 48.81%), no male hypospadiac fetuses were identified in the control group (Table 2). Figure 1 shows the morphologic observations and findings of histological examination of GT in male rat fetuses at GD19.

**TABLE 2:** Effects of DBP on hypospadias rates of male fetal rats

	DBP	Control
No. of pregnant rats	14	11
Total fetuses	181	131
No. of male fetuses	84	67
No. of female fetuses	97	64
No. of hypospadiac male fetal rats (%)	41(48.81)*	0(0)

* Significantly different from control, *p* < .05.

### 3.2 LncRNA NONRATT008453.2 was the highest expressed autophagy‐related lncRNA

**FIGURE 2:**
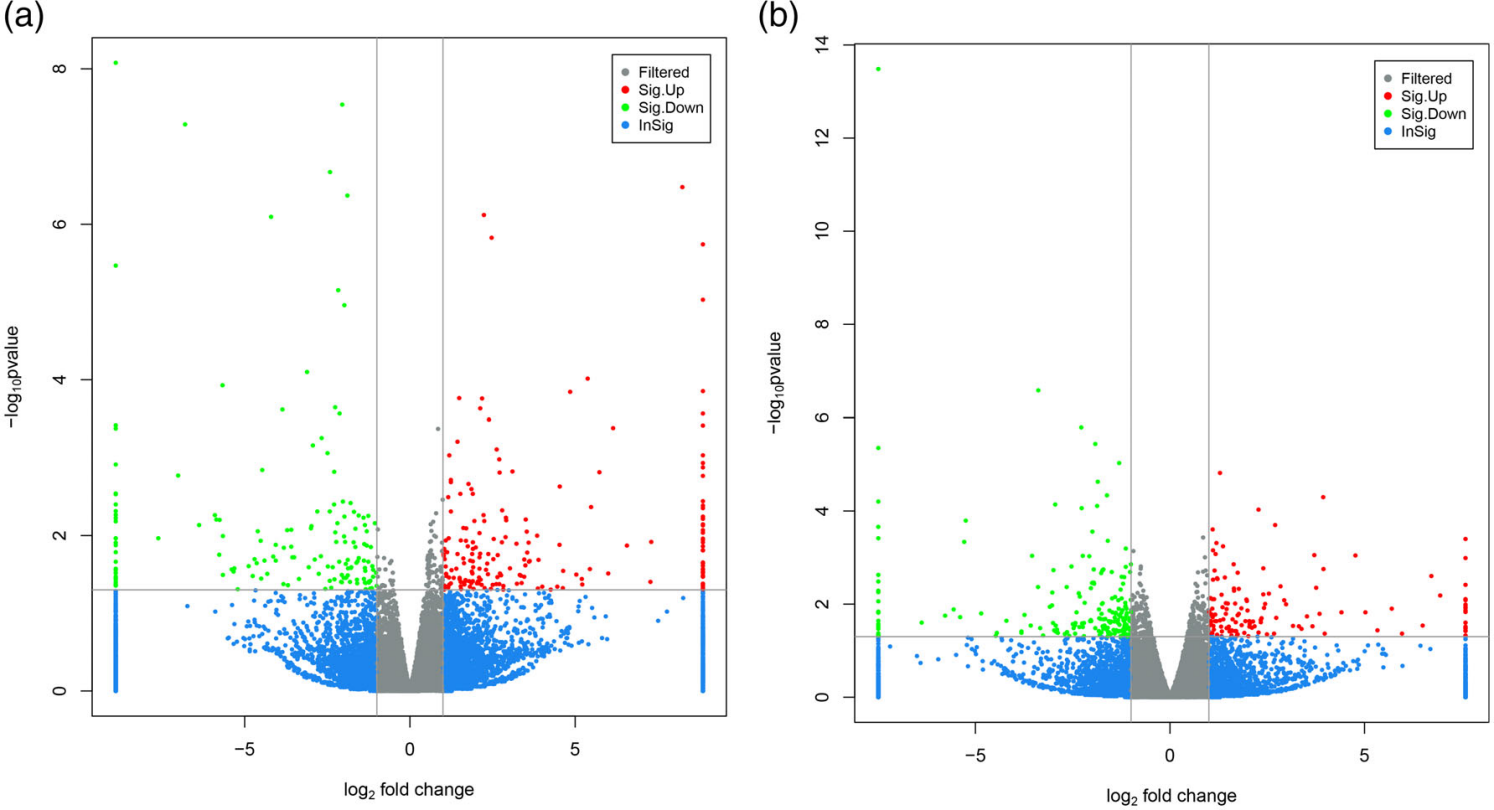
Differential expression of lncRNAs (a) and the mRNA (b) expression changes in the GTs of hypospadiac fetuses show a Benjamini–Hochberg false discovery rate, corrected *p <* .05 compared with the controls; absolute fold change >1.5; (*n* = 3). The volcano plot illustrates the significantly downregulated and upregulated genes. NONRATT008453.2 is an upregulated lncRNA with fold change of 2.3 (*p* < .05)

Sequencing results are illustrated as volcano plots. Comparing hypospadiac fetuses of the same litter (*n* = 3 litters) in the DBP‐treated group to fetuses of the same litter (*n* = 3 litters) in the control group, there were 598 differentially expressed mRNAs; of these, 269 were upregulated and 329 were downregulated. In addition, 427 differentially expressed lncRNAs were identified between the two groups; of these, 253 were upregulated and 174 were downregulated (Figure 2). Interestingly, according to function prediction, 103 differentially expressed lncRNAs were predicted to be related to autophagy. Furthermore, out of the 103 putative autophagy‐related lncRNAs, NONRATT008453.2 was the highest expressed lncRNA in the DBP‐treated group compared with the controls. NONRATT008453.2 has a length of 657 nucleotides located on chromosome 13.

### 3.3 Validation of the sequencing results by qRT‐PCR

**FIGURE 3:**
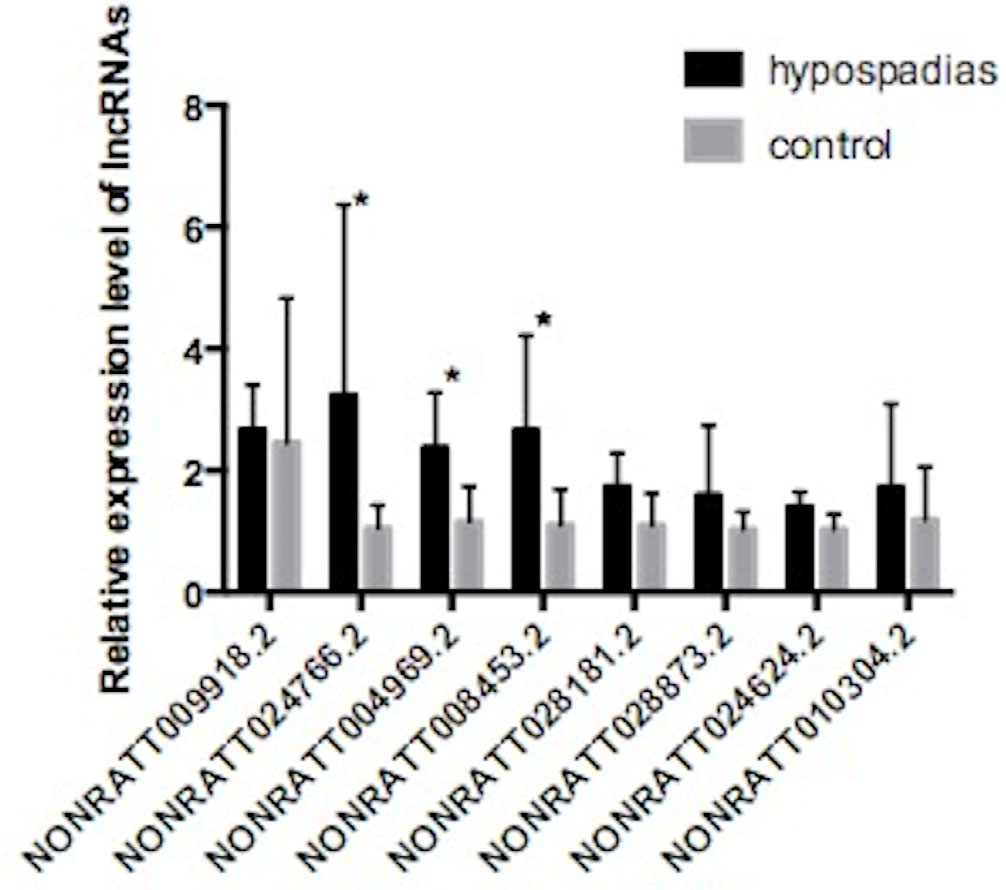
The differential expression levels in GTs between hypospadiac fetuses and normal fetuses detected by qRT‐PCR assay (*n* = 4). NONRATT24766.2, NONRATT004969.2, and NONRATT008453.2 upregulated in hypospadias group compared with the control group (*p* < .05). The expression level of NONRATT009918.2, NONRATT028181.2, NONRATT28873.2, NONRATT24624.2, and NONRATT010304.2 were no difference between hypospadias group and control group (*p* > .05)

To validate the sequencing results, we selected eight lncRNAs according to the bioinformatics analysis and measured the differential expression in GTs between hypospadiac fetuses and normal controls using the qRT‐PCR method. We selected four GTs of male hypospadias fetal rats randomly from different litter in the DBP group and four GTs of male rats from the control group (one from each of four litters) to detect the expression level of eight lncRNAs by qRT‐PCR. The trend of the relative changes of the eight lncRNAs verified with qRT‐PCR is shown in Figure 3.

### 3.4 Identification of the GT fibroblasts

**FIGURE 4:**
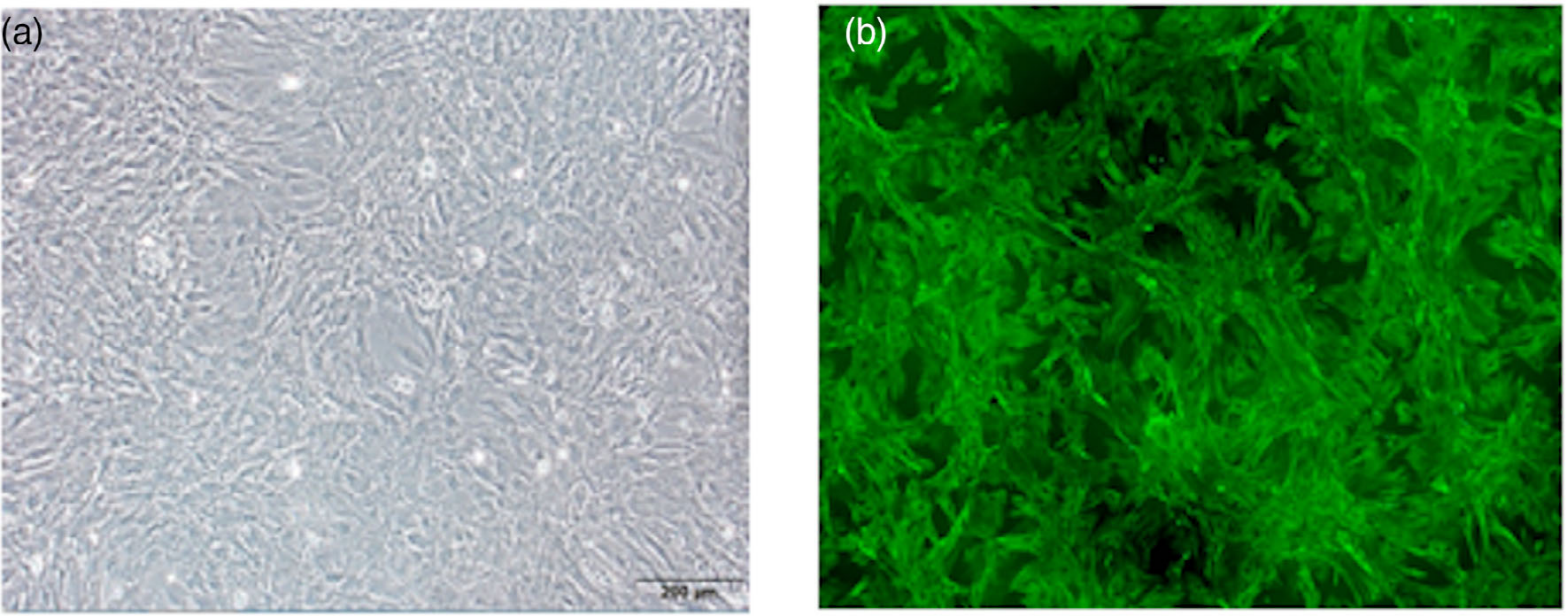
(a) The morphology and distribution of GT fibroblasts from male rat fetuses. (b) The fibroblasts stained positive for vimentin (magnification: ×100)

The GT fibroblasts were successfully separated and cultured by a series of primary cell culture assays according to the cell morphology and the expression of specific cell marker. The GT fibroblasts from male rats were long, spindle‐shaped, and appeared like a palisade or whirlpool under an inverted microscope (Figure 4a). Vimentin is commonly present in mesenchymal cells and was positively expressed in the GT fibroblasts, as determined by immunofluorescence (Figure 4b).

### 3.5 Overexpression of NONRATT008453.2 significantly suppressed fibroblast proliferation

**FIGURE 5:**
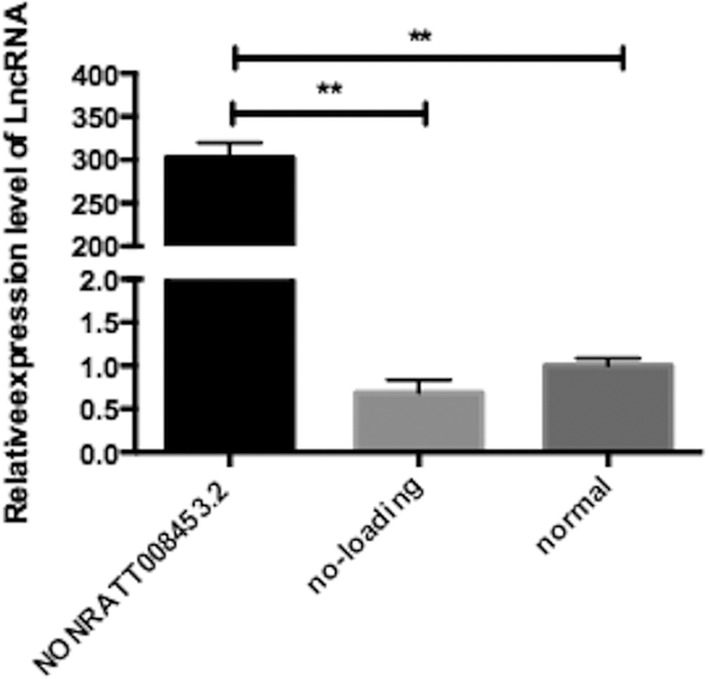
The relative expression level of NONRATT008453.2 increased significantly in the NONRATT008453.2 overexpression group (*p* < .05); however, there was no difference between the no‐loading group and the normal group (*p* > .05)

**FIGURE 6:**
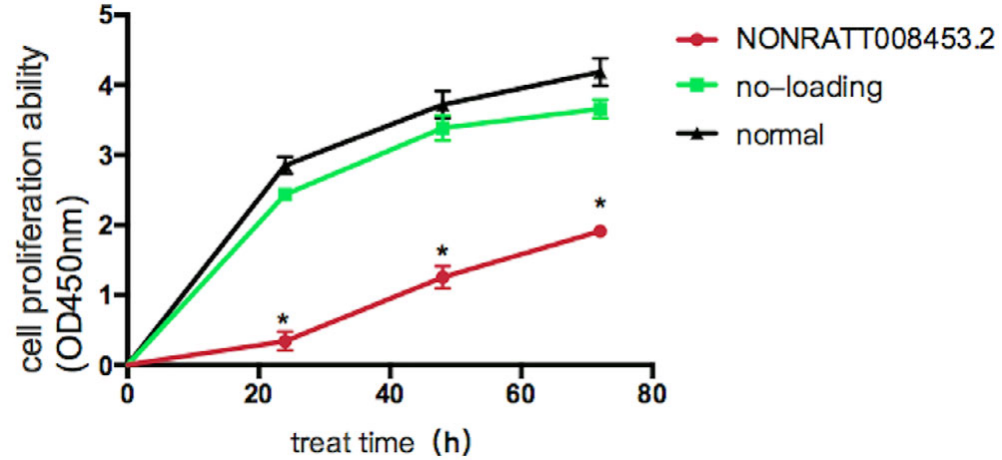
The proliferative capacity of fibroblasts overexpressing NONRATT008453.2 was lower than the other groups at 48 and72 hrs (*p* < .05). There was no significant difference between the no‐loading group and the normal group with respect to proliferative capacity

The GT fibroblasts from hypospadias male rat fetuses were infected with the adenovirus containing NONRATT008453.2 or empty virus alone. The infection efficiency is about 70% (multiplicity of infection, MOI = 20). The morphology of fibroblasts after treatment showed no significant difference compared with normal fibroblasts. The results of qRT‐PCR showed that NONRATT008453.2 was significantly overexpressed in NONRATT008453.2‐overexpressing GT fibroblasts (Figure 5). The results of the CCK‐8 assay demonstrated that proliferation of fibroblasts was significantly suppressed by NONRATT008453.2 overexpression (Figure 6).

### 3.6 Overexpression of NONRATT008453.2 suppressed autophagy in GT fibroblasts from hypospadiac male rat fetuses

**FIGURE 7:**
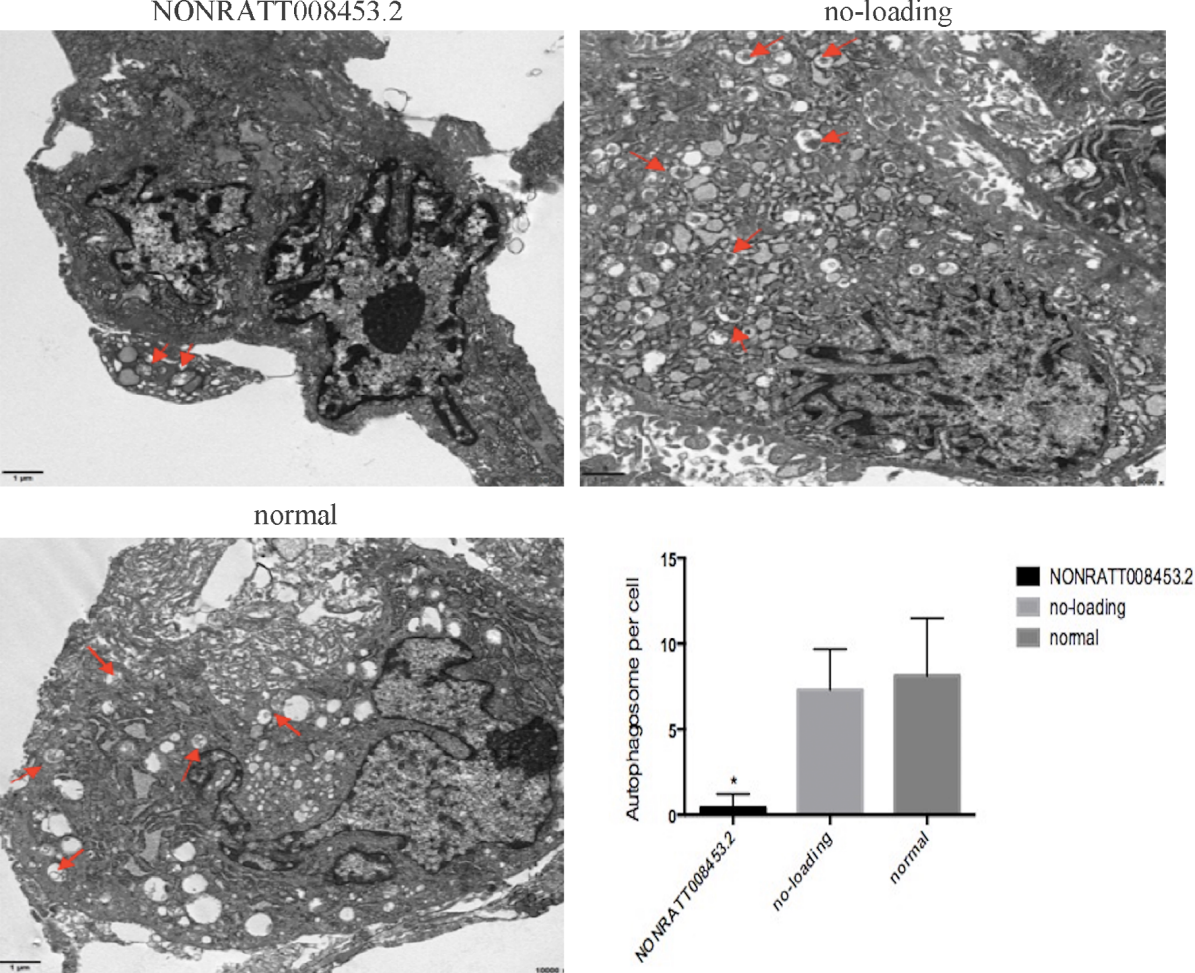
The effects of NONRATT008453.2 overexpression on autophagy in GT fibroblasts from hypospadias rats under a TEM. The red arrow refers to the autophagic lysosome formed by the fusion of autophagosome and lysosome, and the autophagosome is a formation with double‐membrane wrapping cytoplasm. The lysosome has a monolayer containing localized cytoplasm or organelles digested by lysosome. The number of autolysosomes and autophagosomes was clearly lower in the NONRATT008453.2 overexpression group (*p* < .05)

Autophagosomes (double‐membrane vesicles) were observed in the fibroblasts under an electron microscope (Figure 7; arrowheads). Comparison between the no‐loading group and the normal group shows that the number of autolysosomes and autophagosomes was clearly lower in the NONRATT008453.2 overexpression group (*p* < .05). The no‐loading group and the normal group showed no significant difference (*p* > .05).

### 3.7 The effects of NONRATT008453.2 overexpression on autophagy‐associated proteins

**FIGURE 8:**
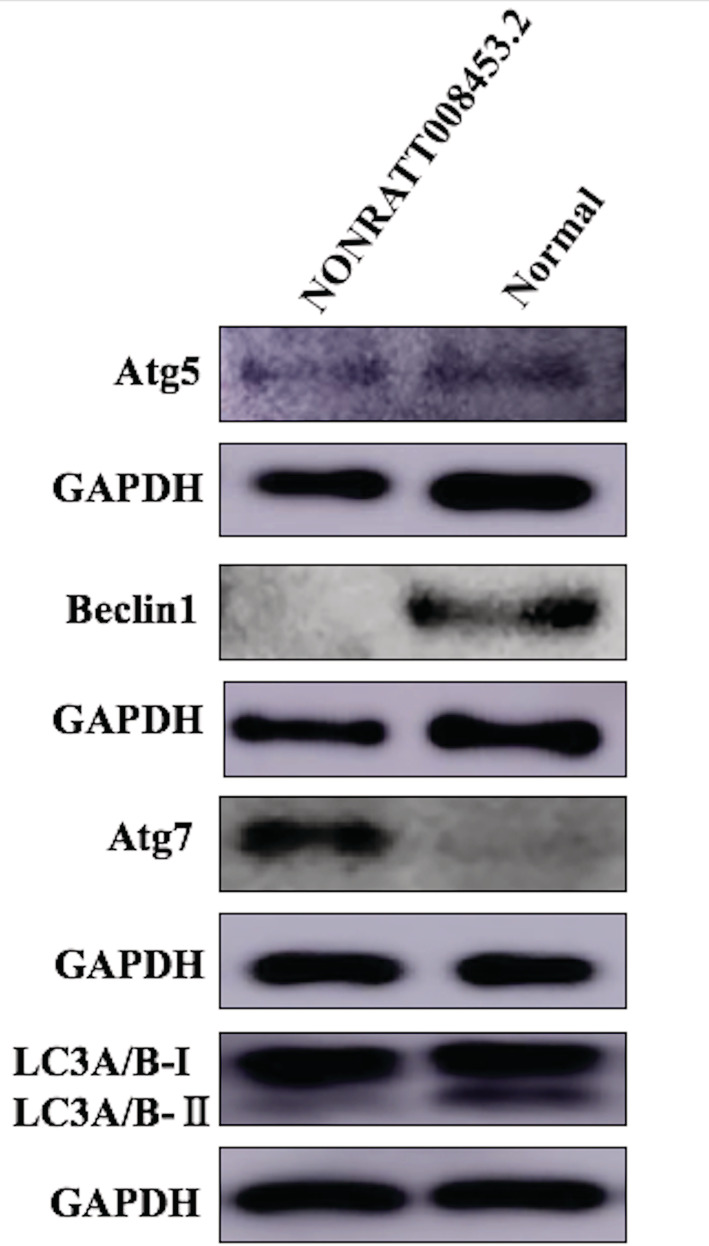
The effects of NONRATT008453.2 overexpression on autophagy‐associated proteins in GT fibroblast from hypospadiac fetus

The expression levels of autophagy‐associated proteins, Atg5, Beclin‐1, Atg7, and LC3, were detected by western blot in the GT fibroblast from hypospadiac male rat fetuses with NONRATT008453.2 overexpression and the normal control cells. As shown in Figure 8, NONRATT008453.2 overexpression markedly promoted the protein expression of Atg7, but inhibited the expressions of Atg5, Beclin‐1, and the ratio of LC3A/B‐II to LC3A/B‐I in the GT fibroblasts.

## 4 DISCUSSION

In recent years, a lot of research has focused on the molecular mechanisms of the pathogenesis and progression of hypospadias to identify potential diagnostic and prognostic biomarkers, as well as therapeutic targets for hypospadias. Hypospadias is believed to be caused by inadequate fusion of the urethral folds and interruption of the continuous growth of the urethral plate into the GT (Baskin, Erol, Li, & Cunha, 1998); this phenomenon can be traced back to embryonic primordial germ cells (PGCs), which later migrate along the genital ridge, where they contribute to the developing gonad (Ginsburg, Snow, & McLaren, 1990). LncRNAs have been shown to participate and play a vital role in some reproductive processes such as embryonic development, ovarian development, and lactation via dealing with hypoxia, hunger, and other physiological stressors (Bertani, Sauer, Bolotin, & Sauer, 2011; Li, Li, Zhang, & Zhou, 2017; Li, Li, Zheng, et al., 2017; Nakagawa et al., 2014; Shore et al., 2012; Yang et al., 2017). LncRNAs may bind to transcriptional factors and activate them to initiate the PGC program by binding to the promoters of downstream target genes to activate or to repress their transcription (Zhang, Gao, & Xu, 2016).

The external genitalia originate from the GT via gathering of mesenchymal cells at the border of the cloacal membrane. Since genital fibroblasts are considered to be the main target cells for androgen and the androgen signaling pathway is closely associated with autophagy in androgen‐sensitive cells, we selected GT fibroblasts as the research objects. Some studies have indicated the role of lncRNA in regulating autophagy, and it regulates several Atg genes at the same time in order to adjust the various stages of autophagy (Xu, Yan, Qian, & Gong, 2017). For instance, MEG3, a new type of tumor inhibitor, which is downregulated in bladder cancer, was shown to exhibit a negative correlation with autophagy markers (Atg8/LC3) (Ying et al., 2013). We supposed that certain lncRNAs may be involved in the pathogenesis of hypospadias in the developing GT by affecting autophagy. According to bioinformatics analysis, we predicted the functions of lncRNA, including biological processes, cellular components, molecular functions, and cellular pathways**.** We found that NONRATT008453.2 showed the highest differential expression among the selected 103 lncRNAs associated with the function of cell autophagy. The overexpression of NONRATT008453.2 in the GT fibroblasts from hypospadias rats directed us to explore how NONRATT008453.2 affects autophagy. To investigate the action of NONRATT008453.2 in GT fibroblasts from hypospadiac male rat fetuses, we analyzed the expression of autophagy protein in fibroblasts from hypospadias GT tissues and normal GT tissues. TEM showed that the overexpression of NONRATT008453.2 inhibits autophagy in the GT fibroblasts from hypospadiac rats. Furthermore, the results of western blot showed that NONRATT008453.2 affects cell autophagy by regulating the expression levels of Atg5, Atg7, Beclin1, and LC3A/B proteins. Of these Atg proteins, the Beclin 1 complex (Beclin 1 is the mammalian ortholog of yeast Atg‐6) and two ubiquitin‐like conjugation systems, the Atg12 and light chain 3 (LC3) systems (LC3 is the mammalian ortholog of yeast Atg8), act sequentially during the nucleation and expansion of the autophagosomal membrane (Scarlatti, Maffei, Beau, Codogno, & Ghidoni, 2008). Studies have shown that autophagy depends on Atg5/Atg7 and is associated with microtubule‐associated protein light chain 3 (LC3) truncation and lipidation (Kang, Zeh, Lotze, & Tang, 2011). These results may indicate a close relationship between NONRATT008453.2 and autophagy in the development of hypospadias. Upregulation of autophagy was observed in fetal GTs after exposure to DBP in utero in rats by inhibiting the PI3k/AKT/mTOR signaling pathway without activation of Beclin‐1 in previous report (Li, Li, Zhang, & Zhou, 2017; Li, Li, Zheng, et al., 2017). However, our present study demonstrated that autophagy was suppressed by overexpression of NONRATT008453.2 in the GT fibroblasts from hypospadiac male rat fetus. One possible explanation is that the effects and the patterns of activating autophagy vary in different cell lines. More studies are needed to explore further mechanisms in which NONRATT008453.2 regulates autophagy and clarify the functions of NONRATT008453.2 participating in the process of histones and chromatin remodeling, transcriptional regulation, and protein–protein interaction (Ernst & Morton, 2013).

## CONFLICTS OF INTEREST

The authors declare no conflicts of interest.

## AUTHOR CONTRIBUTIONS

Xiao Feng, Enfu Huang, Ya Zhang, and Yun Zhou participated in the conception and design of the study. Xiao Feng, Enfu Huang, and Yuanyuan Gao performed the experiments. Xiao Feng analyzed the data. Xiao Feng wrote the manuscript.

## Data Availability

All data generated or analyzed during this study are included in this published article.
